# Tumour-Derived Cell Lines and Their Potential for Therapy Prediction in Patients with Metastatic Colorectal Cancer

**DOI:** 10.3390/cancers13184717

**Published:** 2021-09-21

**Authors:** Sandra Wagner, Nicola T. Beger, Stephanie Matschos, Antonia Szymanski, Randy Przybylla, Florian Bürtin, Friedrich Prall, Michael Linnebacher, Christina S. Mullins

**Affiliations:** 1 Molecular Oncology and Immunotherapy, Department of General Surgery, University Medicine Rostock, 18057 Rostock, Germany; sandra.wagner@med.uni-rostock.de (S.W.); Nicola.Beger@med.uni-muenchen.de (N.T.B.); Stephanie.Matschos@med.uni-rostock.de (S.M.); antonia.szymanski@uni-rostock.de (A.S.); Randy.Przybylla@med.uni-rostock.de (R.P.); florian.buertin@med.uni-rostock.de (F.B.); christina.mullins@med.uni-rostock.de (C.S.M.); 2 Institute of Pathology, University Medicine Rostock, 18057 Rostock, Germany; friedrich.prall@med.uni-rostock.de

**Keywords:** colorectal cancer, metastasis, chemosensitivity, therapy prediction, matching cell line pairs of primaries and metastases

## Abstract

**Simple Summary:**

Colorectal cancer is a global issue with millions of patients in need for new treatment options. Surgical resection is often the first clinical intervention and can be, especially for primary tumours, curative. However, advanced tumours, recurrences, and metastases require drug treatment. Our aim was to compare material of primary tumours and corresponding metastases to draw conclusions from the first one to the latter. The feasibility of therapy prediction using the primary tumour material for the metastatic situation creates a time window for functional testing, which can realistically be integrated into the strict timeframe of clinical procedures. A reliable treatment recommendation, based on the tumour drug response, would improve treatment success in patients with metachronous metastases and spare them from unnecessary side effects of unsuccessful therapies.

**Abstract:**

The prognosis of metastatic colorectal cancer (CRC) remains poor. Patients and physicians are in need of individual therapies and precise response predictions. We investigated the predictive capacity of primary tumour material for treatment response of metastases. Mutational landscapes of primary tumours and corresponding metastases of 10 CRC patients were compared. Cell line characteristics and chemosensitivity were investigated pairwise for primary and metastatic tumours of four patients. PDX models of one patient were treated in vivo for proof of concept. Driver mutations did not differ between primaries and metastases, while the latter accumulated additional mutations. In vitro chemosensitivity testing revealed no differences for responses to 5-FU and oxaliplatin between primary and metastatic cell lines. However, irinotecan response differed significantly: the majority of metastases-derived cell lines was less sensitive to irinotecan than their matching primary counterpart. Therapy recommendations based on these findings were compared to clinical treatment response and mostly in line with the predicted outcome. Therefore, primary tumour cell models seem to be a good tool for drug response testing and conclusion drawing for later metastases. With further data from tumour-derived cell models, such predictions could improve clinical treatment decisions, both recommending likely effective therapeutic options while excluding ineffective treatments.

## 1. Introduction

Colorectal cancer (CRC) is one of the most commonly diagnosed cancers, with 1.4 million new cases annually and almost 700,000 deaths worldwide [1]. At the time of diagnosis, around 20% of CRC patients present with synchronous metastases and up to 60% develop distant metastases within five years [2]. Treatment of CRC is composed of surgical resection and/or medical treatment, whereas solely surgery is curative only for a smaller amount of CRC patients, especially with early tumour stages. The majority of patients are in need of pharmacological therapy, in most cases starting with a variety of chemotherapeutics as first line treatment.

These agents can be applied in a neoadjuvant or adjuvant setting and even in cases of advanced tumour progression or in the metastatic situation. A common first-line chemotherapeutic agent for CRC is fluorouracil (5-FU) which is often applied in combination with oxaliplatin and/or irinotecan to increase response rates [3]. Even though the triple chemotherapy can increase the response rate from 50% [4] to 70% [5,6], there is still a substantial group of patients not benefitting from this treatment. Especially the treatment of metastases urgently needs improvement as patients with distant metastases face a five-year survival of only 14% compared to 90% for patients with local disease [7]. Furthermore, drug resistance, intrinsic or acquired, is observed in around 90% of patients with metastatic CRC [8]. Therefore, it would be of the utmost interest to draw conclusions from the patient’s primary tumour and predict the sensitivity of the metastasis towards different (chemo)therapeutic substances.

To further this individualised therapeutic approach, we established patient-derived cancer models from primary tumours as well as matched metastases from CRC patients with different tumour locations, molecular subtypes and mutational profiles. Either patient tumour material or patient-derived xenograft tissues were used to start the cell line establishment process [9].

We could successfully establish cell line pairs of primary carcinomas and their corresponding metastases from seven different CRC patients. In one of those cases, we were even able to generate a cell line out of the primary tumour, a synchronous as well as a metachronous metastasis. Four of these cell line pairs were analysed in detail with regard to growth behaviour, molecular pattern and chemosensitivity. We studied the response of these matching cell lines side-by-side to the first-line chemotherapeutics 5-FU, oxaliplatin and irinotecan in vitro. For the set consisting of primary, synchronous and metachronous model, we additionally analysed drug responses in PDX models in vivo.

The aim of the study was to unveil commonalities and differences between primaries and metastases. And more importantly, we explored the feasibility of drawing conclusions from primary tissue characteristics (growth behaviour, chemosensitivity etc.) to metastasis. This option would pave the way for predicting treatment success for metastases using the primary material and thus save precious time in the clinical context.

## 2. Materials and Methods

### 2.1. Cell Line Establishment

Cell lines were established as described in detail before [9]. In short, primary CRC resection specimens or corresponding metastases were received fresh from surgery, with informed written patient consent. All procedures were approved by the Ethics Committee of the University of Rostock University Medical Centre (Reference numbers: II HV 43/2004 and A 45/2007) in accordance with generally accepted guidelines for the use of human material. A single cell suspension was generated using crossed scalpels and passing of tissue through a 100 µm cell strainer. Cells were then seeded on a collagen-coated 6-well plate and continually growing cells were passaged and stocked regularly. This process was applied to patient tumour samples as well as PDX samples. Cells were routinely tested for mycoplasma contamination.

Names of the cell lines consist of the following information: Place of material collection (HRO = Hanse City of Rostock), tumour entity (C = Colon), patient number. In the case of a collected metastasis, “Met” is added after the patients’ ID, and in the case of xenograft-derived cell lines number of tumour transfer in mice (e.g., T1) and mouse identifier (e.g., M2) are included in the cell line name.

### 2.2. Mice and Tumour Xenografting

PDX were established as described in detail before [10]. Under specified pathogen-free conditions mice breeding was done in the animal facility of the Rostock University Medical Center. All experiments were performed according to the guidelines of the local animal use and care committee (Landesamt für Landwirtschaft, Lebensmittelsicherheit und Fischerei Mecklenburg-Vorpommern, permit number: LALLF M-V/TSD/7221.3-1.1-098/12). Experiments were performed on approximately 6–8-week-old female and male NMRI-*Foxn1*^nu^ mice (NMRI nu/nu, *n* = 72) weighing 18–22 g. Mice received standard pellet food and water ad libitum. Tumour pieces from previously xenografted patients’ tumours with less than 5 mouse transfers were used and implanted subcutaneously into the animals’ right flank under anaesthesia (ketamine/xylazine, 90/6 mg/kg bw). Tumour growth was regularly monitored, and therapy was initiated upon tumour establishment (ca. 65 mm^3^). Tumour size was measured using a slide scale, and tumour volume was calculated with the following formula:

(1) $$\frac{{\mathrm{tumour}\;\mathrm{length}*\mathrm{tumour}\;\mathrm{width}}^{2}}{2}.$$

### 2.3. Chemosensitivity In Vivo

Mice with solid tumours (65 mm^3^) were randomised into four treatment groups: (1) 5-FU (30 mg/kg bw) (2) irinotecan (20 mg/kg bw) (3) FIRI (5-FU 30 mg/kg bw and irinotecan 20 mg/kg bw) (4) control group receiving equal amount of 0.9% NaCl solution. All therapeutic regimens were applied i.p. thrice weekly for a total of 6 injections. Tumour growth was monitored thrice weekly, within therapy as well as during one week of follow up time. Termination criteria were weight loss >20% vs. start of therapy, impaired socio-physiological behaviour, or tumour sizes >1500 mm^3^. For detailed examinations, tumour tissues were resected, dissected, and cryopreserved as well as embedded in paraffin. H&E sections (4 µm) were prepared for histopathological examinations, according to standard protocols for surgical pathology reports. In a blinded study, morphology of tumour architecture, cytological features, and signs of therapy-induced necrosis/apoptosis of all obtained PDX material was examined and scored (0: no histological response to therapy and 5: very strong therapy response) by an expert pathologist (F.P.).

### 2.4. Chemosensitivity In Vitro by Crystal Violet Staining

Tumour cells (10–15,000 cells/well) were seeded in triplicates in 150 µL/well standard medium in a flat 96-well plate and allowed to attach overnight. Then, chemotherapeutics were added in 50 µL medium yielding the following final concentration ranges: (1) 1 nM–5000 µM 5-FU; (2) 0.1 nM–1000 µM irinotecan; (3) 1.3 nM–20 µM oxaliplatin and (4) FIRI. Medium exchange was performed after 72 h with the addition of fresh chemotherapeutics. After a second 72 h treatment period, viability of the cells was measured.

Wells were washed with PBS to remove dead cells and fragments before the staining. For 96-well plates, 50 µL/well crystal violet staining solution was used. After 20 min incubation at room temperature, wells were washed three times with PBS and dried. To solubilize the colorant, 100 µL of 1% sodium dodecyl sulfate solution was added and incubated for 10 min on a plate shaker. Measurement of absorbance at 590 nm was performed using a Tecan Infinite 200 Pro plate reader and percentage of living cells was determined as follows:

(2) $$\mathrm{Viability}=\frac{\mathrm{OD}\left[\mathrm{sample}\right]-\mathrm{OD}\left[\mathrm{blank}\right]}{\mathrm{OD}\left[\mathrm{living}\;\mathrm{control}\right]-\mathrm{OD}\left[\mathrm{blank}\right]}.$$

IC50 values were calculated using GraphPad Prism 5 and heatmaps were created using TBtool [11].

### 2.5. Colony Formation Assay

After the preparation of a single cell suspension 100 cells/well were seeded in quadruplets in a 96-well plate in 200 µL standard medium. Outgrowing colonies were stained after 14–20 days with crystal violet as described above and counted manually.

### 2.6. Spheroid Formation Assay

After the preparation of a single cell suspension, 100,000 cells/well were seeded in a 6-well plate for suspension cell culture in 5 mL defined serum free medium. Spheroid formation was evaluated qualitatively after 7–14 days and photographically documented.

### 2.7. Next Generation Sequencing Analyses

Tumour DNA was isolated from PDX samples snap frozen in liquid nitrogen by using the Precellys Tissue DNA Kit (PEQLAB Life Science, Erlangen, Germany). Quality criteria for DNA included a concentration ≥25 ng/µL and a DNA integrity number ≥5. Next Generation Sequencing was than performed by Centogene (Rostock, Germany) applying the augmented Solid Tumour Panel including 105 fully sequenced genes plus mutational hot spots from additional 146 genes (see Table S1). A coverage of ≥200× at >97% of targeted regions was reached with a specificity of >99.9% for all reported variants. Sensitivity of single nucleotide variants detection was ≥5% allele frequency. Pathogenicity classification was verified by an automated query to ClinVar (https://www.ncbi.nlm.nih.gov/clinvar/, accessed on 28 August 2021) followed by a manual evaluation.

Concerning the NGS data, for this study, we focused on mutations classified as either pathogenic or likely pathogenic as well as mutations of uncertain significance. Excluded were all synonymous and benign as well as likely benign mutations. Quality criteria included a variant allele frequency of at least 25.

## 3. Results

### 3.1. Patient Characteristics

We collected tumour and corresponding metastatic material of 10 patients (three female and seven male) aged 44–82 years (mean 68.7 years; median 74 years). Detailed patient characteristics and clinical information can be depicted from Table S2.

TNM classification of the primary tumours revealed medium and high-grade adenocarcinomas (T2, T3 and T4) classified to UICC III (3 patients) or IV (7 patients). The majority of metastases were found in the liver (9), but three patients developed metastases either in the peritoneum (2) or in the brain (1) (see Table 1). Six patients showed synchronous metastases whereas three patients were diagnosed with metachronous metastases years after the initial diagnosis. One patient displayed synchronous as well as metachronous metastases (HROC277).

Most tumours and metastases were untreated when resected. Only one patient (HROC348) received neo-adjuvant irradiation and only HROC277Met2 was drug-treated before metastatic resection (see Table 1 and Table S2). Following excision of the tumour, patients were treated with the following compounds: 5-FU, irinotecan, oxaliplatin, bevacizumab, cetuximab and trifluridine/tipiracil (for detailed information, see Table S2).

### 3.2. Molecular Characterisation of Tumour Material

The ten analysed patient tumours’ molecular subtype could be classified as sporadic standard CRC (6/10), CpG island methylator phenotype high and microsatellite stable CRC (3/10) as well as sporadic microsatellite instable CRC (1/10) [12].

The analysis of genetic aberrations in tumours and metastases revealed a relatively low number of mutations detected in the selected panel. This included 251 tumour relevant genes. After application of quality and exclusion criteria we detected 30 genetic aberrations (1–6 per sample, see Table S3). Of the 30 detected non-synonymous mutations, 27 were single base substitutions and 3 were deletions/insertions.

**Figure 1:**
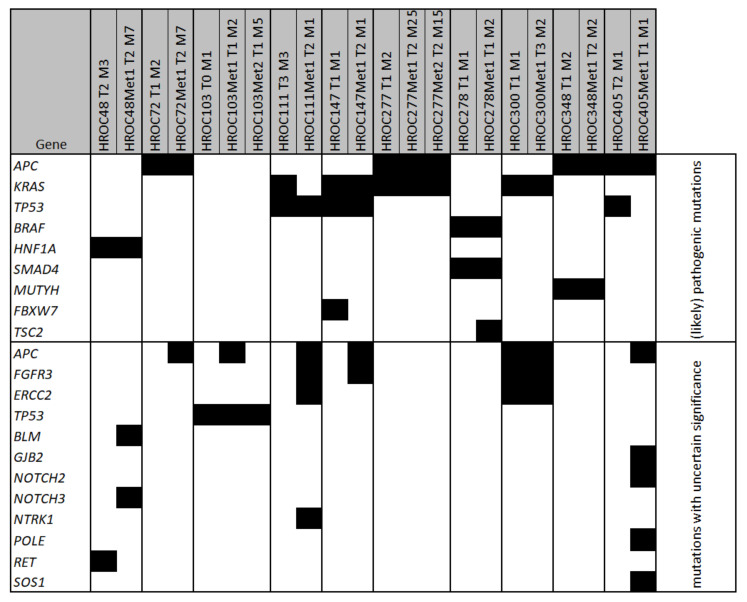
Mutated genes in samples of patient-derived xenografts. Depicted are the genes with ≥1 detected mutation (marked as black boxes) in the Solid Tumour Panel consisting of 105 fully sequenced genes plus mutational hot spots from additional 146 genes with an allele frequency ≥25. Xenograft names consist of patient ID (HROCxxx), number of transfers (Tx) and mouse identifier (Mx).

In four of the ten cases, the number of mutations did not vary between tumour and metastasis, whereas six pairs presented with more mutations in the metastasis. The tumour and Met2 of HROC103 showed one mutation while the Met1 had an additional mutation. There were commonalities as well as differences between tumours and metastases regarding the mutated genes. Comparing their mutational profile, three sets of tumour and metastases were similar, whereas the detected mutations of seven patients differed between tumour and metastasis (see Figure 1). We found in both material sets mutations in nine genes (*APC*, *KRAS*, *TP53*, *ERCC2*, *FGFR3*, *BRAF*, *HNF1A*, *MUTYH*, *SMAD4*) including well-known driver mutations like G12D in the oncogene *KRAS* and truncating mutations of the tumour suppressor gene *APC*. Furthermore, there were two genes (*FBXW7*, *RET*) mutated exclusively in tumours and eight (*BLM*, *GJB2*, *NOTCH2*, *NOTCH3*, *NTRK1*, *POLE*, *SOS1*, *TSC2*) solely in metastases. Especially in the metastases, there was an increase of mutations classified as ‘uncertain significance’, which represent more than 40% of all detected alterations.

Focusing on (likely) pathogenic mutations, we identified 33 mutations in nine genes (0–3 mutations per sample). The most frequently mutated genes were *APC*, *KRAS* and *TP53*. Mutations in the *APC* gene were detected in the samples of four patients, which showed all individual mutations (see Table S3). Notably, four of these mutations were terminating mutations and one was a frameshift mutation. *KRAS*, a typical mutation site in CRC, was mutated in four patients individually: G12D, G12V, G12A and G13R. Furthermore, (likely) pathogenic mutations were detected in *BRAF*, *HNF1A*, *SMAD4*, *MUTYH*, *FBXW7* and *TSC2.* Pathogenic and likely pathogenic mutations were mainly consistent in tumours and metastases. Interestingly, HROC103 showed only mutations of uncertain significance and no (likely) pathogenic mutation was detected.

### 3.3. Morphology and Cell Doubling Time of Selected Cell Lines

For in vitro experiments, cell line pairs of the following patients were selected based on feasibility criteria (especially growth patterns and cell doubling times) and further characterised: HROC147, HROC277, HROC278, and HROC348. For an overview, see Table 2.

HROC147 T0 M1 was established from a xenograft, whereas the cell line of the corresponding metastasis HROC147Met1 originates directly from patient material. Cells of both cell lines grew in dense, clearly outlined round colonies limited in size and did not form a confluent monolayer. Doubling times of HROC147 T0 M1 and HROC147Met1 were 63 h and 58 h, respectively.

The cell lines of patient HROC277 differed in their morphology as well as their proliferation rate. HROC277 T0 M1 and HROC277Met2 appeared to grow in colonies without contact inhibition, leading to the establishment of a confluent monolayer. On the other hand, HROC277Met1 T0 M2 favoured growth in single colonies not spreading over the culture vessel. The lowest proliferation rate in this set of cell lines was observed for HROC277Met1 T0 M2 with a doubling time of 66 h. The doubling times of HROC277 T0 M1 and HROC277Met1 T0 M2 were similar with 62 h.

The xenograft-derived matching cell lines of patient HROC278 were dissimilar in their cultivation appearance. The cell line of the tumour, HROC278 T0 M1, formed small and dense colonies with miniature adjacent colonies. HROC278Met1 T2 M2 grew in bigger and less dense colonies. According to their morphology, their rate of proliferation differed, too. The cell line of the metastasis, HROC278Met1 T2 M2, grew slightly faster than the tumour-derived cell line (54 h versus 61 h).

HROC348 and the corresponding metastasis HROC348Met1 were directly patient-derived cell lines. They both favoured growth on collagen-coated cell culture vessels and built similar irregularly shaped colonies that were characterised by small cells. Doubling times of HROC348 and HROC348Met1 were 74 h and 66 h, respectively.

### 3.4. Colony Formation and Spheroid Formation

All cell lines used were tested positive in the colony formation assay, thereby proving their capacity to form colonies out of single cells (see Table 2). A significant difference between tumours and their metastases was only seen in two sets of cell lines. The number of colonies formed by HROC277Met1 T0 M2 was significantly lower than for its matching tumour counterpart (*p* < 0.001). The opposite was observed in the cell line pair of HROC348. Here, the metastasis-derived cell line formed significantly more colonies than the tumour (*p* < 0.001).

In the spheroid formation assay, most tested cell line pairs grew into spheroids with no morphological differences between tumour-derived and metastasis-derived cell lines. Only the spheroids of HROC278 T0 M2 and HROC278Met1 T2 M2 did not resemble each other. HROC278 T0 M2 formed spheroids of irregular shape with dark inclusions, whereas spheroids of HROC278Met1 T2 M2 were round and of light colour.

### 3.5. Chemosensitivity In Vitro

**Figure 2:**
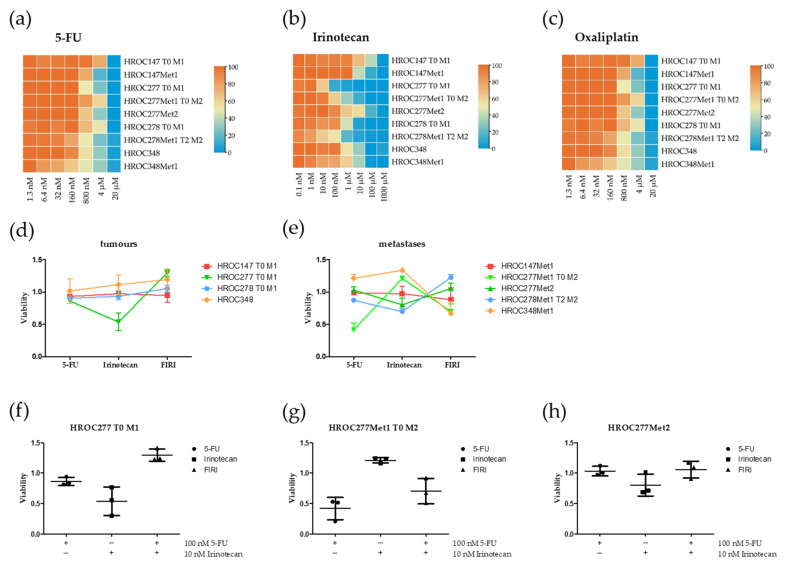
Viability of CRC cell lines after treatment with 5-FU (5-Fluorouracil), irinotecan and oxaliplatin in vitro. Cell lines were treated for 7 days with (**a**) 0.1 nM–5000 µM 5-FU, (**b**) 0.1 nM–1000 µM irinotecan or (**c**) 1.3 nM–20 µM oxaliplatin. Cell lines were treated with low doses of 5-FU, irinotecan and FIRI (**d**,**e**). The cell lines (**f**) HROC277 T0 M1, (**g**) HROC277Met1 T0 M2 and (**h**) HROC277Met2 were treated for 7 days with 100 nM 5-FU, 10 nM irinotecan or 100 nM 5-FU + 10 nM irinotecan (FIRI). Viable cells were measured in triplicates with crystal violet assay in three independent experiments. For the calculation of viability, the optical density of treated cells was normalised to untreated cells. Results are depicted as mean with standard deviation. Detailed information on cell line nomenclature can be found in material and methods section. Tx: Number of transfers; Mx: Mouse identifier.

The in vitro chemosensitivity testing revealed 5-FU sensitivity of all tested cell lines (see Figure 2A). IC_50_ values ranged from 0.5 to 9.2 µM (see Table 3) and notable differences of IC_50_ values could be detected for matching cell line pairs of HROC147 and HROC277. Here, IC_50_ values of the metastatic cell lines were decreased compared to the tumour cell lines but without reaching significance. The 5-FU IC_50_ values of HROC278 and HROC348 did not differ between tumour- and metastasis-derived cell lines.

A remarkable difference was observed for the responses to irinotecan (see Figure 2B). HROC147Met1 showed a significantly higher IC_50_ than HROC147 T0 M1 (IC_50[HROC147 T0 M1]_ = 0.8 µM; IC_50[HROC147Met1]_ = 2.2 µM; *p* < 0.05). The results for the cell lines from case HROC277 were even more diverse. HROC277Met2 was least responsive (IC_50[HROC277Met2]_ = 15.6 µM; *p* = 0.02 versus HROC277 T0 M1), followed by HROC277Met1 T0 M2 (IC_50[HROC277Met1 T0 M2}_ = 9.2 µM; *p* = 0.05 versus HROC277 T0 M1) and HROC277 T0 M1, which was relatively sensitive (IC_50[HROC277 T0 M1]_ = 0.1 µM).

Further, HROC348 and its matching metastasis-derived cell line tended to behave similar (IC_50[HROC348]_ = 2.1 µM; IC_50[HROC348Met1}_ = 16.8 µM; *p* = 0.08). In summary, in three of four clinical cases, the metastasis showed lower chemosensitivity to irinotecan than the primary tumour in vitro.

The range of IC_50_ for oxaliplatin was 2.0–4.9 µM (see Table 3). Metastatic cell lines of HROC147, HROC278 and HROC348 showed a slightly higher sensitivity against oxaliplatin than their corresponding primary tumour cell lines (see Figure 2C). However, the metastatic cell lines of HROC277 responded differently. HROC277Met1 T0 M2 had a lower IC_50_ than the primary tumour cell line, whereas HROC277Met2′s IC_50_ was significantly higher than the primary tumour and the synchronous metastasis cell lines.

In summary, all tested cell lines reacted sensitive to 5-FU, irinotecan and oxaliplatin and no intrinsic resistance could be observed.

Combinations of the used chemotherapeutics in low doses did not lead to a synergistic effect but to additive effects at best (Figure 2D–H). Exemplarily, the results of HROC277 are depicted in Figure 2F–H. In this set of cell lines, the effects of 5-FU and irinotecan even seem to annihilate each other.

### 3.6. Patient HROC277

For a proof of concept study in vivo, one patient set (HROC277) was selected since this case includes a synchronous and a metachronous metastasis and PDX models are available for all three specimen (primary tumour and both metastases). The 77-year-old male patient underwent preventive colonoscopy when a tubular adenocarcinoma protruding from the orifice of vermiform appendix was detected. A right hemicolectomy with en bloc resection of an infiltrated intestinal loop was performed and intraoperatively a singular metastasis of hepatic segment III was completely resected. Histopathological analyses revealed two large moderately differentiated adenocarcinomas of the appendix vermiformis with extramural venous invasion, but without involvement of the regional lymph nodes. A complete resection of the singular liver metastasis (G2 pT4a (2) pN0(0/12) L0 V1 pM1 (liver) R0; UICC IV A) was successful. Adjuvant chemotherapy was implemented with four courses of 5-FU, folinic acid, and oxaliplatin (FOLFOX4) and continued with seven courses of 5-FU and folinic acid due to elevated creatinine. Fourteen months later, re-staging by computed tomography and magnetic resonance imaging revealed a large metachronous metastasis of the right liver lobe. After two courses of 5-FU, folinic acid, irinotecan (FOLFIRI) and bevacizumab, the patient underwent anatomical resection of the hepatic segments VI, VII and VIII with histopathological confirmation of complete resection. Yet, nine months later, multiple pulmonic and hepatic metastases were detected by computed tomography and palliative treatment was continued with three courses of FOLFIRI and bevacizumab. The patient succumbed to his disease 39 months after the initial diagnosis.

### 3.7. Chemosensitivity In Vivo

**Figure 3:**
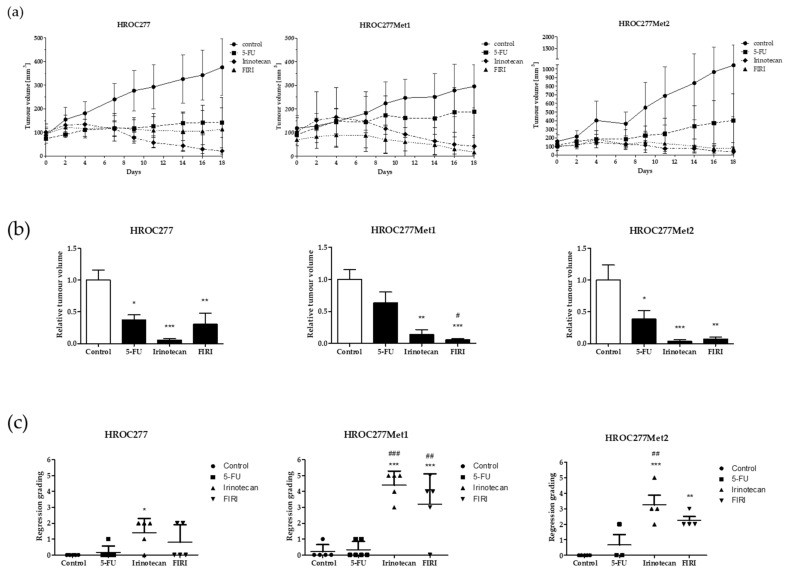
Chemosensitivity in vivo. Xenografted mice were treated with 5-FU (30 mg/kg bw), irinotecan (20 mg/kg bw), FIRI (5-FU 30 mg/kg bw and irinotecan 20 mg/kg bw) or 0.9% NaCl solution in the control group. Tumour volume was measured under therapy (**a**) and at day 18 (**b**). Regression grading (**c**) was performed by an experienced pathologist indicating 0: no histological response to therapy and 5: very strong therapy response. Results are depicted as mean with standard deviation. Statistical results are based on an ANOVA test indicating *p*-value <0.05 (* or #), *p*-value <0.01 (** or ##) and *p*-value <0.001 (*** or ###). * treatment group vs. untreated control group; # irinotecan- or FIRI-treated mice vs. 5-FU-treated mice. Bw: body weight.

Xenografted and tumour-bearing mice received 5-FU, irinotecan, the combination (FIRI) or 0.9% NaCl solution in the control group. Therapy with 5-FU resulted in decreased tumour growth in all treated mice with significant tumour shrinkage in HROC277 and HROC277Met2 (see Figure 3A). However, the effect of 5-FU was considerably exceeded by the treatment with irinotecan. In HROC277- and HROC277Met2-PDX, irinotecan reduced the tumour volume by 94% and 96% compared to the control, respectively (see Figure 3B). Only in HROC277Met1-PDX the effect of irinotecan was even surpassed by FIRI, resulting in tumour volume reduction of 94% (compared to irinotecan treatment alone: 86%). The regression grading was performed by an experienced pathologist and revealed that irinotecan, in all three cases, led to a significantly increased grade of regression compared to control and also to 5-FU-treated mice (see Figure 3C). A benefit from FIRI compared to irinotecan treatment could not be observed.

## 4. Discussion

Our group aimed at establishing models from CRC patients (cell lines and PDX) for investigating commonalities as well as differences in genetic profiles and drug responses between primary and metastatic lesions of the same patient on the cellular level. Moreover, we aimed at functionally testing the predictive capabilities of primary tumour models for the treatment of metastases.

The side by side comparison of primary and metastatic tumour samples from 10 CRC patients revealed a relatively low number of detected mutations in tumour as well as metastasis. The metastases had thereby the same or an increased number of relevant mutations compared to the tumours. Furthermore, (likely) pathogenic mutations mainly coincided in the paired samples and were even observed in different patients. This is in line with results from a study analysing primary CRC as well as matching liver metastasis, where also no significant differences regarding copy number variations or mutations leading to deregulation of signalling pathways could be found [13].

Hu and colleagues described concordant driver mutations between primary tumour and metastasis pairs of 25 CRC patients [14]. They also developed a model for phylogenetic reconstruction of metastatic CRC. In most patients, dissemination of cancer cells was an early process so that tumours as well as metastases have ample time to accumulate additional private mutations. This also explains the ‘absence’ of certain mutations in metastases, which were present in the primary tumour. Moreover, the increased number of mutations of ‘uncertain significance’ mainly present in metastases provides further evidence for this concept of early dissemination and independent metastases development.

In contrast to our results, a large study including almost 1200 CRC patients, found the tumour mutational burden in metastases lower as compared to the primaries [15]. The authors explained this with the greater likelihood of hypermutated tumours to be eliminated by immune mechanisms [16,17]. Divergence between mutational analyses, including our results, can, at least partly, be explained by the heterogeneity of a given tumour. Individual tumours are typically composed of a variety of cellular clones, all harbouring their unique mutational pattern. The piece of tumour material used for genetic analysis and/or tumour model generation represents a fragment of the tumour, rather than reflecting the whole landscape of genetic alterations [18,19]. A detailed description of our CRC PDX model biobank will be published soon. There, we will also cover the genetic characteristics of our tumour model collection (*n* = 125 CRC patients/PDX models) containing the patients’ models included in the present manuscript.

In this study we could detect (likely) pathogenic mutations in nine genes, among them common CRC-related mutation sites in *APC* [20,21], *KRAS* [22,23], *BRAF* [24,25] *SMAD4* [26,27] and *TP53* [28,29]. *HNF1A*, which was found mutated in one patient, is a transcription factor regulating cell proliferation, migration, invasion, and colony formation in CRC [30]. Another gene affected by mutations was FBXW7 which is, according to experimental approaches, involved in epithelial-mesenchymal-transition and chemo-resistance [31]; while a different study revealed no strong association between patient prognosis and FBXW7 mutations [32].

Mutations in *MUTYH* were observed to be predispositions and can increase the risk of developing CRC as biallelic germline mutations are associated with hereditary polyposis [33]. In our analysis, we could detect a heterozygous mutation in primary as well as metastatic tissue of the case HROC348.

As the genetic analyses did not reveal substantial differences between primary tumours and metastases, conclusions drawn from the primary tumour composition should permit reliable predictions. We thus hypothesised that drug responses might not differ significantly between primary tumours’ and metastatic cells from individual cases. In order to test this, functional assays were performed in vitro. Cell lines in low passages, i.e., between passage 20–30, were used as these still recapitulate the intrinsic neoplastic characteristics well [34].

Four cell line pairs were selected and characterised regarding morphology, growth behaviour, cloning and spheroid formation capacity as well as sensitivity to different therapeutics in vitro. Morphological differences between cell lines from different patients were observed but only minor variations in cell line sets. The cell lines reached doubling times between 54 h and 74 h, comparable to established and well-characterised cell lines like Caco-2 (doubling time 60–80 h [German Collection of Microorganisms and Cell Culture; Cell Lines Service]).

When treating the cell lines with the clinical first line chemotherapeutics 5-FU, irinotecan as well as oxaliplatin, we observed high sensitivity. 5-FU treatment resulted in distinct anti-tumour effects and IC_50_ values were well below the reachable plasma concentration of 380 µM for 5-FU [35]. Furthermore, IC_50_ values were similar or even decreased in metastasis cell lines compared to their matching primary, indicating high predictive capability of both tumour model types for 5-FU.

Oxaliplatin was also able to eradicate the tumour cells independently of primary or metastatic origin in a dose (IC_50_: 2–5 µM) matching the reachable plasma concentrations of oxaliplatin, which has been found to be 3–8 µM [36,37,38]. Thus, a therapy including oxaliplatin is supported by these data, albeit it seems likely that therapy will select for resistant subclones due to the proximity of reachable plasma concentrations and IC_50_. In a study testing extreme drug resistance with chemotherapeutic doses 5–80-fold higher than those achieved clinically, it could also be shown that the response of tumour and metastatic cells of CRC patients did not differ significantly [39]. This effect was observed for 5-FU, irinotecan and oxaliplatin, but in this study unmatched tumours and metastases were analysed.

Our in vitro chemosensitivity testing showed the most diverse results for irinotecan. All tested CRC cell lines reacted to the treatment and no intrinsic resistance was observed (acquired resistance under long term treatment was not tested). But the metastasis-derived cell lines of three patients were less sensitive towards irinotecan than the tumour cell lines. This is in accordance with results from Takebayashi et al. who also observed lower sensitivity, especially in liver metastatic cells compared to paired CRC primary tumour cells when treating with irinotecan, but not with 5-FU or oxaliplatin [40]. In the cell lines HROC277Met2 (IC_50[HROC277Met2]_ = 15.59 µM) and HROC348Met1 (IC_50[HROC348Met1]_ = 16.8 µM), the IC_50_ exceeded the normal plasma level of irinotecan (up to 2 µM [41]) by far; suggesting a likely failure in clinical therapy.

We further tested if combinations of the chemotherapeutics 5-FU and irinotecan act synergistically in lower doses. Contrarily to what was expected, viability decreasing effects of irinotecan were abrogated by 5-FU.

Exemplarily, we selected an in vivo PDX model set of one CRC patient from whom we had PDX models of the primary tumour (HROC277), the synchronous (HROC277Met1) and the metachronous (HROC277Met2) metastases for validation of these in vitro results. All PDX models were treated with 5-FU, irinotecan and their combination FIRI. The results confirmed that a combinational approach with these chemotherapeutic substances is not superior to the best-acting single chemotherapy, i.e., irinotecan; and thus corroborating the in vitro results of HROC277. In contrast, the different responsive behaviours of the metastasis-derived cell clones compared to the primary-derived ones under irinotecan treatment obtained in vitro could not be reproduced. One explanation for this phenomenon could be the comparatively low activity of irinotecan compared to its hydrolysed compound SN-38 [42]. Metabolisation of the prodrug is catalysed by carboxylesterases mainly found in the liver and also in normal colon tissue but to a lower extent in CRC cells [43]. Therefore, while reaching relatively low plasma levels (maximal concentration of approximately 3–15 µM in the mouse [44]), the concentration of the highly active compound SN-38 can be increased in vivo compared to in vitro resulting in higher chemosensitivity.

Based on these in vitro and in vivo results, we retrospectively stated therapy recommendations and compared them with the actual clinical treatment success of the patients. For patient HROC147, we would have proposed chemotherapy including 5-FU and oxaliplatin. Indeed, FOLFOX (5-FU+leucovorin+oxaliplatin) therapy led to a complete remission of the patient.

Patient HROC277 underwent surgical resection of the tumour and its synchronous metastasis before chemotherapy started with FOLFOX, followed by FOLFIRI. The metachronous metastasis HROC277Met2, detected under FOLFIRI treatment, was resected subsequently. But the following FOLFIRI cycles did not result in tumour control and the disease progressed with further metastases. Our in vitro studies match these potential treatment failures with relatively high IC_50_ values for irinotecan. The promising results from the in vivo experiments probably trace back to the much higher plasma level in the mouse [44] compared to the patient in clinical treatment. Furthermore, regarding sensitivity to 5-FU and irinotecan, the synchronous metastasis behaved more like the metachronous metastasis than like the primary tumour. Therefore, it should be considered to also include synchronous metastases into the data sets used for further treatment predictions of later developing metastases. This could also have helped patient HROC348, who presented a synchronous metastasis with the highest IC_50_ value of all tested cell lines for irinotecan. With this knowledge, the patient could have been spared from irinotecan-containing treatments including their side effects and other promising therapy options should have been used in first-line treatment. Even the primary tumour presented a rather high IC_50_ for irinotecan in the range of reachable plasma levels, indicating potential treatment difficulties.

The cell lines of HROC278 were highly sensitive for 5-FU and irinotecan and intermediate for oxaliplatin proposing an excellent treatment success. Nevertheless, FOLFIRI plus bevacizumab therapy after resection failed to prevent the development of a further metastasis, and the patient died 26 months later. As a likely explanation, the relatively high tumour burden due to the incomplete resection may have caused rapid disease progression by proliferation of chemo-resistant subclones.

## 5. Conclusions

The initial question of this study was whether it is possible to predict biological behaviour of metastases by concluding results obtained from CRC cells of the primary tumour. As an ideal tool to answer this question, the cell line pairwise comparison confirmed that both phenotype and growth behaviour of CRC cells did not differ significantly between tumour- and metastasis-derived cells. Also, the chemosensitivity tests revealed no significant differences between tumour and metastasis for the majority of cell line pairs and tested substances. Consequently, we would conclude that analysing and testing cells from primary CRC allows for identifying promising treatments for metastases likely to develop in the future. Besides approaching chemo response with 2D cell cultures, patient-derived organoids (PDOs) delivered also encouraging results. Studies investigating PDOs from either tumour or metastatic material could confirm a correlation between PDO reactivity and patient’s response to chemotherapy [45,46]. However, despite the fact that our study is the first using four tumour and metastatic cell line pairs, more such data have to be collected in terms of individual patient tumour model pairs. Furthermore, the comparison of untreated and pretreated tumours and metastases regarding their therapeutic sensitivities could help to improve treatment success predictions. Similar to the classical chemotherapeutics analysed in the present study, further therapeutic substances, including preclinical drug candidates, must and can be surveyed taking advantage of our cell line pairs, which are available for other researchers upon reasonable request.

## Figures and Tables

**Table 1:** Characteristics of tumours and metastases.

Tumour ID	Collection Site	Metastasis Appearance	Pretreatment	Molecular Subtype
HROC48	Transverse Colon	-	no	spMSI
HROC48Met1	Multivisceral (small intestine, stomach, pancreas)	metachronous (56 months)	no	-
HROC72	Transverse Colon	-	no	spStd
HROC72Met1	Liver	synchronous	no	-
HROC103	Rectum	-	no	spStd
HROC103Met1	Liver	metachronous (48 months)	no	-
HROC103Met2	Liver	metachronous (67 months)	no	-
HROC111	Left Colon	-	no	spStd
HROC111Met1	Brain	metachronous (30 months)	no	-
HROC147	Sigmoid colon	-	no	CIMP-H, non-MSI
HROC147Met1	Liver	synchronous	no	-
HROC277	Right colon/Appendix	-	no	spStd
HROC277Met1	Liver	synchronous	no	-
HROC277Met2	Liver	metachronous (14 months)	yes	-
HROC278	Right colon	-	no	CIMP-H, non-MSI
HROC278Met1	Peritoneum	synchronous	no	-
HROC300	Rectum	-	no	CIMP-H, non MSI
HROC300Met1	Liver	synchronous	no	-
HROC348	Sigmoid	-	yes	spStd
HROC348Met1	Liver	synchronous	yes	-
HROC405	Right colon	-	no	spStd
HROC405Met1	Liver	synchronous	no	-

CIMP: CpG island methylator phenotype; ID: identification number; MSI: microsatellite instable; spMSI: sporadic microsatellite instable, spStd: sporadic standard.

**Table 2:** Cell Line Characterisation. Pictures were taken at 10× magnification with a light microscope.

Cell Line	2D Cell Culture	3D Cell Culture	Doubling Time (h)	Colony Formation (%)
HROC147 T0 M1	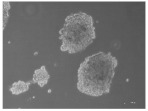	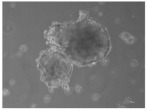	62.6 (±4.2)	6.7 (±4.0)
HROC147Met1	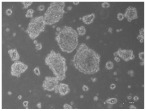	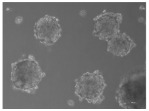	57.8 (±3.2)	5.5 (±2.2)
HROC277 T0 M1	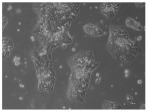	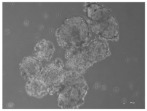	61.6 (±11.3)	17.6 (±5.1)
HROC277Met1 T0 M2	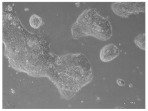	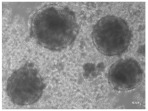	66.3 (±3.6)	3.3 (±1.7)
HROC277Met2	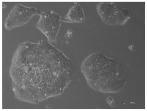	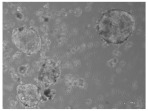	61.6 (±3.7)	11.9 (±3.0)
HROC278 T0 M1	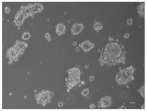	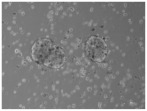	61.0 (±9.8)	3.2 (±3.1)
HROC278Met1 T2 M2	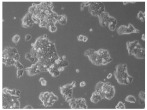	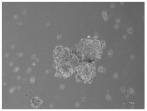	54.1 (±4.4)	4.2 (±4.1)
HROC348	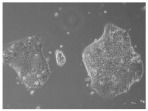	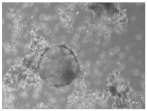	74.2 (±7.2)	1.1 (±1.5)
HROC348Met1	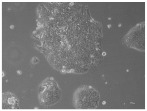	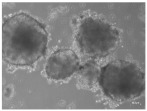	66.1 (±6.7)	3.2 (±2.3)

Detailed information on cell line nomenclature can be found in material and methods section. Tx: Number of transfers; Mx: Mouse identifier.

**Table 3:** IC_50_ values of colorectal cancer (CRC) cell lines. Calculations were based on chemosensitivity in vitro assays (*n* = 5–8 biological replicates consisting of technical triplicates).

Cell Line	5-FU [µM]	Irinotecan [µM]	Oxaliplatin [µM]
HROC147 T0 M1	2.5	0.8	4.5
HROC147Met1	0.5	2.2	3.9
HROC277 T0 M1	9.2	0.1	2.9
HROC277Met1 T0 M2	1.6	9.2	2.6
HROC277Met2	3.7	15.6	4.9
HROC278 T0 M1	3.2	0.8	4.1
HROC278Met1 T2 M2	3.2	0.3	2.8
HROC348	4.4	2.1	2.2
HROC348Met	4.2	16.8	2.0

## Data Availability

The data and materials are available from the corresponding author on reasonable request.

## Supplementary Materials

The following are available online at https://www.mdpi.com/article/10.3390/cancers13184717/s1, Table S1: Solid Tumor Panel and Hotspots for Sequencing, Table S2: Patient information, Table S3: Single Nucleotide Variants of Sequenced CRC PDX.
